# The layout measures of micro-sprinkler irrigation under plastic film regulate tomato soil bacterial community and root system

**DOI:** 10.3389/fpls.2023.1136439

**Published:** 2023-03-08

**Authors:** Mingzhi Zhang, Na Xiao, Haijian Yang, Yuan Li, Fangrong Gao, Jianbin Li, Zhenxing Zhang

**Affiliations:** ^1^ Faculty of Engineering, Huanghe Science and Technology University, Zhengzhou, China; ^2^ Institute of Water Resources and Rural Water Conservancy, Henan Provincial Water Conservancy Research Institute, Zhengzhou, China; ^3^ Vegetable station, Northwest Land and Resources Research Center, Shaanxi Normal University, Xi’an, China; ^4^ Hydraulic Research Laboratory, Yellow River Hydrologic Survey Planning and Design Co., Ltd., Zhengzhou, China; ^5^ Agricultural Technology Extension Center of Xi’an City, Xi’an, Shaanxi, China; ^6^ Key Laboratory of Vegetation Ecology, Ministry of Education, Northeast Normal University, Changchun, China; ^7^ State Environmental Protection Key Laboratory of Wetland Ecology and Vegetation Restoration, School of Environment, Northeast Normal University, Changchun, China

**Keywords:** rhizosphere soil, soil bacteria, root system, yield, positive interaction

## Abstract

**Introduction:**

The change in rhizosphere soil bacterial community and root system under new water-saving device is not clear.

**Methods:**

A completely randomized experimental design was used to explore the effects of different micropore group spacing (L1: 30 cm micropore group spacing, L2: 50 cm micropore group spacing) and capillary arrangement density (C1: one pipe for one row, C2: one pipe for two rows, C3: one pipe for three rows) on tomato rhizosphere soil bacteria community, roots and tomato yield under MSPF. The bacteria in tomato rhizosphere soil were sequenced by 16S rRNA gene amplicon metagenomic sequencing technology, the interaction of bacterial community, root system and yield in tomato rhizosphere soil was quantitatively described based on regression analysis.

**Results:**

Results showed that L1 was not only beneficial to the development of tomato root morphology, but also promoted the ACE index of tomato soil bacterial community structure and the abundance of nitrogen and phosphorus metabolism functional genes. The yield and crop water use efficiency (WUE) of spring tomato and autumn tomato in L1 were about 14.15% and 11.27%, 12.64% and 10.35% higher than those in L2. With the decrease of capillary arrangement density, the diversity of bacterial community structure in tomato rhizosphere soil decreased, and the abundance of nitrogen and phosphorus metabolism functional genes of soil bacteria also decreased. The small abundance of soil bacterial functional genes limited the absorption of soil nutrients by tomato roots and roots morphological development. The yield and crop water use efficiency of spring and autumn tomato in C2 were significantly higher than those in C3 about 34.76% and 15.23%, 31.94% and 13.91%, respectively. The positive interaction between soil bacterial community and root morphological development of tomato was promoted by the capillary layout measures of MSPF.

**Discussion:**

The L1C2 treatment had a stable bacterial community structure and good root morphological development, which positively promoted the increase of tomato yield. The interaction between soil microorganisms and roots of tomato was regulated by optimizing the layout measures of MSPF to provide data support for water-saving and yield-increasing of tomato in Northwest China.

## 1 Introduction

Soil microorganisms are the main components of terrestrial ecosystems, which plays a key role in the decomposition of organic matter, nutrient cycling and the degradation of harmful substances, and also play an important role in facility planting agricultural ecosystems (Zhu et al., 2019; Ma et al., 2023). Soil bacteria are the main component of soil microorganisms (Wang et al., 2017), which have high diversity, high abundance and complete functions (Li and Ma, 2018; Xu et al., 2022; Zhu et al., 2022). Soil bacteria are mainly involved in the formation of humus and the mineralization of organic matter, which is essential for regulating soil enzyme activity, soil nutrient cycle and crop root morphological development (Özbolat et al., 2023; Zheng et al., 2023). Previous studies have found that soil bacterial diversity and heterogeneity are often used to characterize soil fertility and predict ecological environment risks (Guo et al., 2018). Soil bacteria are significantly affected by physical and chemical properties such as soil water, heat and nutrients, plant growth, and other microorganisms (Huang et al., 2018; Zhang et al., 2018). Therefore, the study of soil bacterial community is of great significance for measuring regional soil productive potential and sustainable development.

Facility Planting agriculture has the characteristics of fast crop growth and high demand for soil water and fertilizer (Nie et al., 2022; Zhao et al., 2022). Measuring the level of soil water and fertilizer has become a hot topic in current research. The nutrients needed for plant growth mainly come from the soil, in which the mineralization of soil nutrients by microorganisms and the absorption of nutrients by plant roots are important links in the nutrient cycle (Jafari et al., 2018; Morio et al., 2022). Previous studies have found that soil nitrogen-fixing bacteria can convert nitrogen in the air into nitrogen sources, promote plant root morphological development to support plant needs, and thus reduce plant nutritional stress (Wang J. et al., 2022). Plant roots can make the plant-soil feedback direction develop in a positive direction. In response to soil drought, plant fine roots can grow into aggregates and open microsites, where oxygen stimulates microbial activity and N release. Root exudates and litter produced by root growth provide energy sources for the growth of soil microorganisms (Veresoglou et al., 2022; Xiao et al., 2023). Therefore, it is urgent to explore the relationship between soil microorganisms and plant roots for the efficient utilization of land resources.

Redistribution of soil moisture, heat and air is closely related to field irrigation management, which directly or indirectly affects the development of soil microbial community and plant root morphological development (Feng et al., 2023; Vera et al., 2023). In order to improve the stability of soil microbial community structure in plant root zone and promote root morphological development, researchers have proposed methods such as microbial inoculation and growth regulator addition (Lewis et al., 2020; Zhou et al., 2023). However, there are many problems in the implementation of the above methods, such as high investment cost and environmental pollution (Boddington and Dodd, 1999; Zhao et al., 2017). At the same time, other researchers have found that it is feasible to optimize soil microbial communities and root morphological development in crop root zones by adjusting crop irrigation management methods, such as changes in field layout measures of tomato drip irrigation that can change soil microbial communities and yield (Sun et al., 2021; Wang et al., 2017). Proper field layout of drip irrigation pipe can promote the morphological development of tomato root system (Wang et al., 2020a; Li et al., 2023). The arrangement of drip irrigation under plastic film indirectly affects the morphological development of crop root system and soil microbial community (Wang et al., 2016; Ye et al., 2016). Therefore, exploring the effects of irrigation management measures on crop soil microorganisms and crop roots has guiding significance for elucidating the relationship between soil microbial community and root system in facility planting agriculture and guiding the water-saving, yield-increasing and quality-improving of facility planting agricultural crops.

At present, the mechanism of the change of crop soil bacterial community and root system regulated by the new water-saving technology of facility planting agriculture under MSPF (Zhang et al., 2020a) is not clear. At the same time, the interaction relationship between soil bacterial community-root system-yield of tomato in greenhouse under MSPF lacks qualitative and quantitative description. Therefore, in this study, greenhouse tomato was used as the research object to explore the response of rhizosphere soil bacteria community, root system and yield of greenhouse tomato under MSPF to the different micropore group spacing and capillary arrangement density of micro-sprinkler pipe. The purpose of this paper is to adjust the interaction between tomato roots system and soil microorganisms by optimizing the layout measures of MSPF through greenhouse experiment and mathematical analysis, and to provide data support for the prediction of soil production potential of facility planting agriculture and the water-saving and yield-increasing of tomato.

## 2 Materials and methods

### 2.1 Experimental site and management

**Figure 1:**
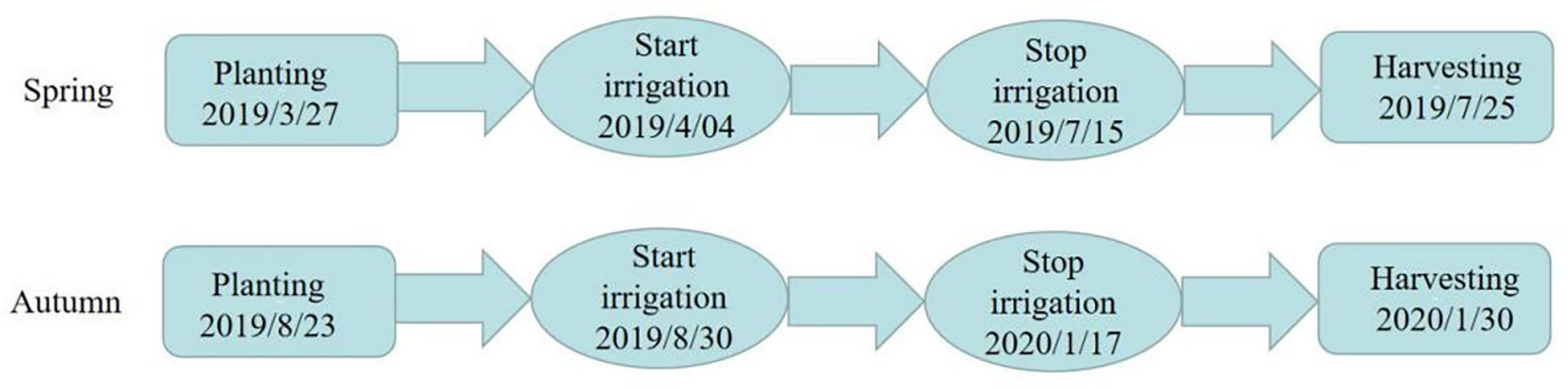
The planting, irrigation and harvesting time of tomato.

**Figure 2:**
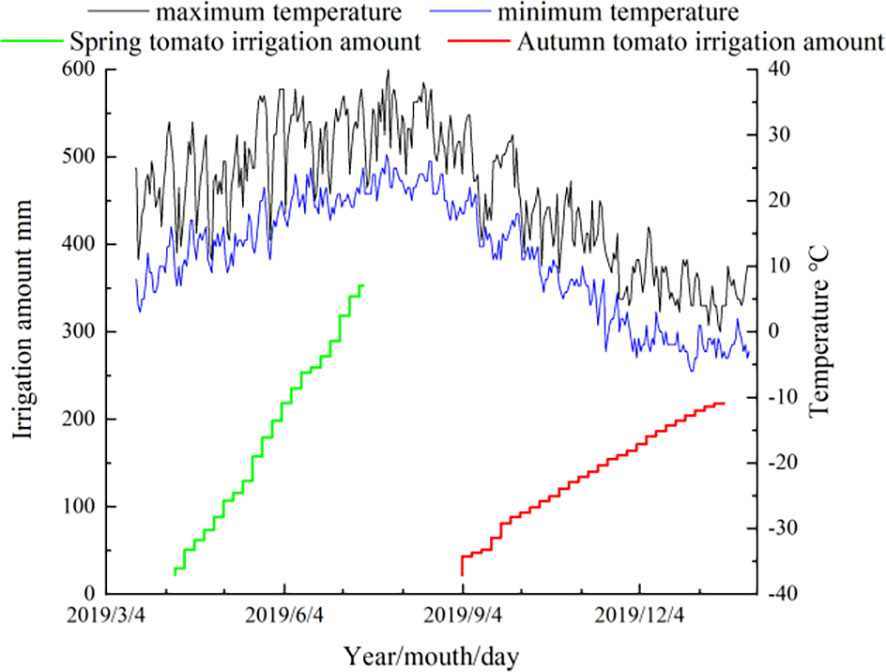
Meteorological data and irrigation record of tomato growth period. Diameter of micropore is d=0.8mm; The internal spacing of the micropores group was I =0.4cm; The Angle of micropores is =68°; The micropore group spacing is L.

The experiment was carried out in the greenhouse of Xi ‘an Modern Agricultural Science and Technology Exhibition Center (108°52’E, 34°03’N) in Shaanxi Province from March 23, 2019 to January 30, 2020. The tested tomato variety was ‘Jingfan 401’ (Jingyan Yinong Seed Industry Technology Co., Ltd., Beijing·China). The tomato adopts the ridge planting structure mode, in which the row spacing is 50.00 cm and the plant spacing is 40.00 cm. The length of the experimental plot is 3.40 m, and the spacing between the plots is 4.00 m. The planting, irrigation and harvest time of spring tomato and autumn tomato are shown in **Figure 1**. Meteorological data and irrigation record of tomato growth period are shown in **Figure 2**.

In this experiment, the irrigation amount was controlled on the basis of the cumulative evaporation from a 20-cm diameter standard pan (Epan, DY.AM3, Weifang Dayu Hydrology Technology Co., Ltd., Shandong, China) following Zhang (Zhang et al., 2020a). The evaporation amount was measured at 08:00 am every 5 d. The irrigation amount was evaluated after the measurement. The irrigation quota was calculated according to Formula (1), and the irrigation times and amounts were recorded (see **Figure 2**).


(1) $$W=A\times E_{pan}\times k_{cp}$$

*E_pan_* represents the evaporation within the interval of two irrigation, based on the cumulative evaporation from a 20 cm diameter pan (mm); *A* represents the capillary control area (mm) and *k*_*cp*_ represents the crop- pan coefficient. In this paper, adopting adequate irrigation mode, the crop-pan coefficient of is 1.0 (Zhang et al., 2020a).

### 2.2 Experimental design

In this experiment, two factors of micropore group spacing and capillary arrangement density were set.

**Figure 3:**
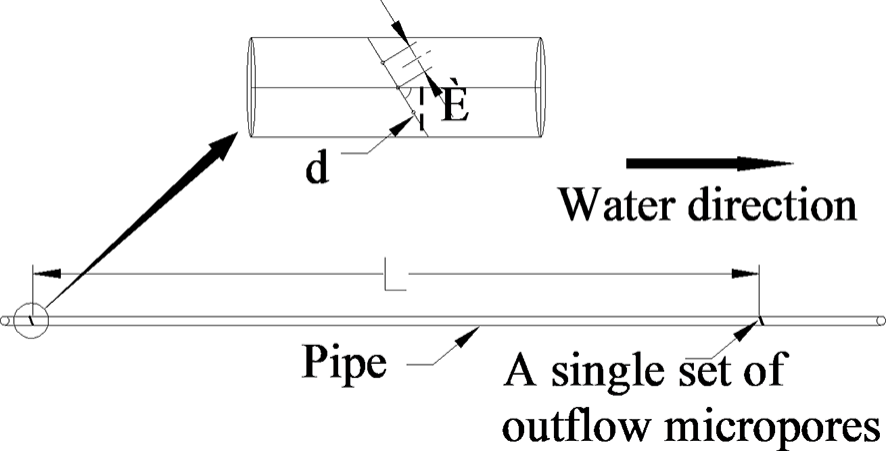
Micropores group (inside) spacing structure parameters (Zhang et al., 2020b). Diameter of micropore is d=0.8mm; The internal spacing of the micropores group was I =0.4cm; The Angle of micropores is =68º; The micropore group spacing is L.

**Figure 4:**
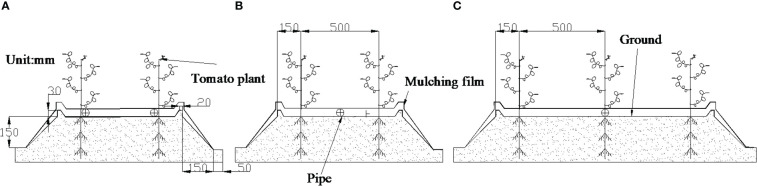
Capillary arrangement. (Zhang et al., 2020b). **(A)** represents one pipe for one row; **(B)** represents one pipe for two rows; **(C)** represents one pipe for three rows.

Micropore group spacing (L, see **Figure 3**) sets 2 levels (30, 50 cm). The capillary arrangement density (C, see **Figure 4**) sets 3 levels ((one pipe for one row, one row of tomatoes irrigated by one pipe, C1), (one pipe for two rows, two rows of tomatoes irrigated by one pipe, C2), (one pipe for three rows, three rows of tomatoes irrigated by one pipe, C3)).

This study consisted of 6 treatments (**Table 1**), each treatment was repeated 3 times, a total of 18 experimental plots. The irrigation water in this experiment comes from the groundwater in the region. The head of the water source is connected in series with a 120-mesh sieve filter. The principle of diversion is used to ensure that the working pressure of the control system is constant, and the complete random test is used to arrange the test fields. Each experimental field was irrigated separately and controlled by a spherical valve.

**Table 1:** Experimental design.

No.	Treatment	Micropore group spacing	Capillary arrangement density	Polt		Tomato growth period irrigation quota mm	
				Spring	Autumn	Spring	Autumn
1	L1C1	30	one pipe for one row	6	6	353.03	218.19
				1	1		
				13	13		
2	L1C2	30	one pipe for two rows	11	11		
				2	2		
				7	7		
3	L1C3	30	one pipe for three rows	16	16		
				10	10		
				5	5		
4	L2C1	50	one pipe for one row	3	3		
				17	17		
				15	15		
5	L2C2	50	one pipe for two rows	18	18		
				9	9		
				4	4		
6	L2C3	50	one pipe for three rows	8	8		
				12	12		
				14	14		

L represents the micropore group spacing, C represents the capillary arrangement density, and one pipe for two rows represents two rows of tomatoes irrigated by one pipe.

### 2.3 Measurements and computational methods

#### 2.3.1 Collection and determination of soil bacterial

1) Soil samples were collected from rhizosphere soil of spring tomato and autumn tomato after 72 days of planting. The soil shaking method was used to extract (Taking tomato plants as the center, the cylindrical soil with tomato roots at a radius of 20 cm and a depth of 5-25 cm was excavated, and the loose soil combined with tomato roots was shaken off. The soil closely combined with tomato roots in greenhouse was gently brushed with a soft brush after sterilization as the rhizosphere soil of tomato in greenhouse). Three rhizosphere soil samples were randomly taken from each experimental plot, and the samples were transported to the laboratory on ice. The samples were brought back to the room and the fresh soil plant residues were removed. Three soil samples in the plot were fully stirred and evenly mixed as soil samples for sequencing in the experimental plot. The soil samples were collected and quickly frozen in liquid nitrogen. The soil samples were stored in a -80°C refrigerator and sent to Shanghai Meiji Biomedical Technology Co., Ltd. (Shanghai, China) to determine soil bacterial community. The main analysis steps of soil bacterial community determination are divided into three parts:

A. DNA extraction and PCR amplification

Total DNA was extracted from tomato rhizosphere soil using the E.Z.N.A.Â ^®^ soil kit (Omega Bio-tek, Norcross, GA, USA), which reliably and quickly separates high-quality genomic DNA from various soil samples (up to 1 g of soil can be processed in 60 min). After the quality of DNA extraction was detected by 1% agarose gel electrophoresis, the V3-V4 variable region was amplified by PCR with 338 F (5 ‘ -ACTCCTACGGGAGGGAGCAGCAG-3 ‘) and 806 R (5 ‘ -GGACTACHVGGGTWTCTAAT-3 ‘) primers. The amplification procedure was: 95 °C pre-denaturation 3 min, 27 cycles (95 °C denaturation 30 s, 55 °C annealing 30 s, 72 °C extension 30 s). Finally, extension at 72 °C for 10 min (PCR instrument: ABI GeneAmp ^®^ 9700).

B. Illumina Miseq sequencing

The bacterial 16S rDNA V3-V4 region was selected, and the NA samples were sequenced using the Illumina Miseq PE300 high-throughput sequencing platform (Shanghai Meiji Biomedical Technology Co., Ltd.). The bacterial 16 S rDNA V3-V4 amplification primers were 338 F (5 ‘ -ACTCCTACGGGGAGGCAGCAG-3 ‘) and 806 R (5 ‘ -GGACTACNNGGGTATCTAAT-3 ‘). The PCR products were recovered using 2% agarose gel, purified and eluted for detection. PCR reaction system (total system was 25 μL): 12.5 μL KAPA 2G Robust Hot Start Ready Mix, 1 μL Forward Primer (5 μmol/L), 1 μL Reverse Primer (5 μmol/L), 5 μL DNA (the total amount of DNA added was 30 ng), and finally 5.5 μL dd H_2_O was added to make up to 25 μL. Reaction parameters: 95 °C for 5 min; 95 °C denaturation 45 s, 55 °C annealing 50 s, 72 °C extension 45 s, 28 cycles; 72 °C for 10 min. The original sequence was uploaded to the figshare database (https://figshare.com/articles/dataset/The_Layout_Measures_of_Micro-sprinkler_Irrigation_under_Plastic_Film_Regulate_Tomato_Soil_Bacterial_Community_and_Root_System/21818610).

C. Sequencing data processing

The original data obtained by Miseq sequencing were optimized after splicing and quality control. After distinguishing the samples, OTU (Operational taxonomic unit) cluster analysis and species taxonomy analysis were performed. The OUT similarity was set to 97%. The OTU was subjected to diversity index analysis and statistical analysis of community structure at each classification level, and then a series of in-depth statistical and visual analysis such as multivariate analysis and difference significance test of sample community composition and phylogenetic information were completed. The ACE index of soil bacterial (ACE), CHAO index of soil bacterial (CHAO), COVERAGE index of soil bacterial (COVERAGE), SHANNON index of soil bacterial (SHANNON), SIMPSON index of soil bacterial (SIMPSON), SOBS index of soil bacterial (SOBS), Species Veen diagram analysis and species composition analysis can be obtained by direct analysis of Shanghai Meiji Biological Cloud platform (https://login.majorbio.com/login).

2) Based on the results of soil bacterial community determination, and referring to the Kyoto Encyclopedia of Genes and Genomes and related references (Wang et al., 2020b), the functional genes related to soil bacterial nitrogen fixation, nitrification, denitrification and phosphorus metabolism functional gene abundance were obtained. The functional genes related to soil bacterial nitrogen metabolism are the sum of the abundance of soil bacterial nitrogen fixation, nitrification and denitrification functional genes.

#### 2.3.2 Root system

Three tomato plants were randomly selected from each plot to dig a soil volume with a depth of about 0.4 m and a diameter of 0.2 m centered on the plant at 76 and 78 days after planting spring tomato and autumn tomato, respectively. The samples were placed in a 150 mesh sieve to rinse the roots. The roots were scanned by Epson Perfection V700 scanner to obtain the TIF diagram. Finally, the TIF diagram was processed by WinRHIZO Pro software to obtain the total root length, total number of root tips and bifurcation number of greenhouse tomatoes. The root activity of tomato was determined by triphenyltetrazolium chloride method (Li et al., 2020).

#### 2.3.3 Yield and water use efficiency

Four tomato plants were randomly selected, and the mature fruit mass of four tomatoes was weighed by electronic scale with precision of 0.01 g, and the yield per hectare was converted.

Time-domain reflectometry soil moisture sensor (TRIME-PICO-IPH, IMKO, Inc., Ettlingen, Germany) was used to measure the soil volume moisture content at different layers of soil (0–10, 10–20, 20–30, 30–40, 40–50, 50–60, 60–70, and 70–80 cm, respectively). It was measured once before and after each growth period. Water consumption (*ET*_*a*_ )and crop water use efficiency (WUE) were calculated formulas (2) and (3), respectively (Zhang et al., 2020a):

(2) $$ET_{a}=I\pm 1000\times H\times \left({\text{θ}}_{t1}-{\text{θ}}_{t2}\right)$$

represents crop water consumption during growth period (mm); *I* represents the irrigation quota of crop growth period (mm); *H* represents the depth of the wetting layer with plan (H = 0.8 m); θ_*t*1_ and θ_*t*2_ represent 80-cm average soil volumetric water contents at times  *t*1 and *t*2 cm^3^/cm^3^), respectively.


(3) $$WUE=1000*\frac{Y}{ET_{a}}\;$$

*Y* indicates crop grain yield (t/hm^2^).

### 2.4 Data analysis

The interaction of bacterial community, root system and yield in tomato rhizosphere soil was quantitatively described based on regression analysis. The significant difference was analyzed by F test of SPSS22.0 (IBM Crop., Armonk, New York, NY, USA), and the significant level was set to *P*<0.05. The picture was drawn by OriginPro2019 (Origin Lab Corporation, Northampton, MA, USA). Excel 2016 (Microsoft Excel, Microsoft, Washington, USA) was used for regression analysis.

### 2.5 Abbreviations

The abbreviations of this article are explained in **Table 2**.

**Table 2:** Abbreviations.

No	Abbreviations	Full name
1	L	Micropores group spacing
2	C	Capillary arrangement density
3	ACE	Ace index of soil bacterial
4	CHAO	Chao index of soil bacterial
5	COVERAGE	Coverage index of soil bacterial
6	SHANNON	Shannon index of soil bacterial
7	SIMPSON	Simpson index of soil bacterial
8	SOBS	Sobs index of soil bacterial
9	NFFA	Soil bacterial nitrogen fixation functional gene abundance
10	NFA	Soil bacterial nitrification functional gene abundance
11	DFA	Soil bacterial denitrification functional gene abundance
12	GAN	Soil bacterial nitrogen metabolism functional gene abundance
13	GAP	Soil bacterial phosphorus metabolism functional gene abundance
14	RL	Total root length
15	RS	Total root surface area
16	RV	Total root volume
17	RT	Total number of root tips
18	RB	Total root bifurcation number
19	RA	Root activity
20	Y	Yield
21	WUE	Water use efficiency

## 3 Results

### 3.1 Effects of different treatments on soil bacterial community of greenhouse tomato

#### 3.1.1 Diversity of soil bacterial community structure

**Figure 5:**
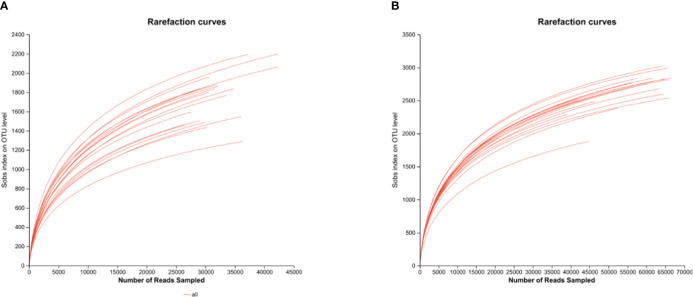
Bacterial dilution curve of tomato rhizosphere soil. **(A)** Spring **(B)** Autumn.

It can be seen from the dilution curve of **Figure 5** that the amount of sequencing data in each treatment is sufficient. It can be seen from **Table 3** that the micropore group spacing (L) had a significant effect on the ACE index of soil bacterial (ACE), CHAO index of soil bacterial (CHAO), SHANNON index of soil bacterial (SHANNON) and SOBS index of soil bacterial (SOBS) of spring tomato and autumn tomato. The relative contribution of L to ACE, CHAO, SHANNON, and SOBS of tomato was 30.20%, 28.80%, 24.20% and 32.80%, respectively. The capillary arrangement density (C) had a significant effect on the ACE, CHAO, SHANNON, SIMPSON index of soil bacterial (SIMPSON) and SOBS of spring tomato and autumn tomato. The relative contribution of C to ACE, CHAO, SHANNON, SIMPSON and SOBS of tomato was 62.60%, 60.40%, 37.50%, 28.30% and 38.4%, respectively. The different planting seasons (D) had a significant effect on the ACE, CHAO, COVERAGE index of soil bacterial (COVERAGE), SHANNON, SIMPSON and SOBS of spring tomato and autumn tomato. The relative contribution of D to ACE, CHAO, COVERAGE, SHANNON, SIMPSON and SOBS of tomato was 62.60%, 60.40%, 37.50%, 28.30% and 38.4%, respectively.

**Table 3:** Diversity of soil bacterial community structure.

	Treatment	ACE	CHAO	COVERAGE	SHANNON	SIMPSON	SOBS
Spring	L1C1	2406.8 ± 29.04ab	2383.97 ± 33.63ab	0.9826 ± 0.00105a	5.92 ± 0.13a	0.0078 ± 0.00094b	1855.67 ± 22.48ab
	L1C2	2652.77 ± 27.34a	2621.97 ± 27.11a	0.9834 ± 0.0032a	5.8 ± 0.42a	0.0162 ± 0.01155ab	2070.33 ± 115.28a
	L1C3	2031.45 ± 74.45cd	2057.69 ± 99.6cd	0.983 ± 0.00138a	5.28 ± 0.15b	0.0219 ± 0.0038a	1528.33 ± 54.72cd
	L2C1	2512.34 ± 256.48ab	2498.21 ± 291.71ab	0.9829 ± 0.00182a	5.7 ± 0.13ab	0.0133 ± 0.0026ab	1950 ± 218.06ab
	L2C2	2283.43 ± 194.19bc	2268.15 ± 165.33bc	0.9844 ± 0.00154a	5.3 ± 0.14b	0.0222 ± 0.00321a	1716.67 ± 150.93bc
	L2C3	1837.32 ± 154.57d	1816.54 ± 173.38d	0.9859 ± 0.00377a	5.28 ± 0.3b	0.0183 ± 0.00758ab	1395.67 ± 94.21d
Autumn	L1C1	3511.24 ± 65.45a	3482.2 ± 55.93a	0.9888 ± 0.00123a	6.38 ± 0.09a	0.0047 ± 0.00069a	2881.67 ± 96.02a
	L1C2	3548.28 ± 52.62a	3531.84 ± 57.54a	0.988 ± 0.00111a	6.26 ± 0.18a	0.0068 ± 0.00219a	2894.67 ± 109.55ab
	L1C3	3228.32 ± 93.53ab	3199.86 ± 122.29ab	0.9858 ± 0.00326a	6.24 ± 0.09a	0.0051 ± 0.00022a	2524 ± 148.93bc
	L2C1	3358.6 ± 137ab	3345.88 ± 126.55a	0.9886 ± 0.00222a	6.19 ± 0.02a	0.0063 ± 0.00056a	2737.33 ± 124.56ab
	L2C2	3218.74 ± 240.75ab	3214.89 ± 253.78ab	0.9876 ± 0.00269a	6.17 ± 0.13a	0.0064 ± 0.00205a	2588.33 ± 139.69abc
	L2C3	2931.81 ± 462.44b	2887.25 ± 474.53b	0.985 ± 0.00224a	6.05 ± 0.38a	0.0069 ± 0.00374a	2261.33 ± 371.27c
F-value	L	10.405** (30.20)	9.687** (28.80)	0.361ns (1.50)	7.665* (24.20)	1.453ns (5.70)	11.738** (32.80)
	C	20.102** (62.60)	18.300** (60.40)	0.539ns (4.30)	7.195* (37.50)	4.714* (28.30)	25.949** (68.40)
	D	250.944** (91.30)	225.566** (90.40)	55.017** (47.80)	87.795** (78.50)	48.827** (67.00)	277.107** (92.00)
	L*C	2.262ns (15.90)	2.235ns (15.70)	0.143ns (1.20)	0.654ns (5.20)	0.825ns (6.40)	2.697ns (18.30)
	L*D	0.700* (2.80)	0.507* (2.10)	1.431ns (5.60)	0.344ns (1.40)	0.282ns (1.20)	0.922ns (40.00)
	C*D	1.165ns (8.90)	0.643ns (5.10)	3.615* (23.20)	2.486ns (17.20)	3.410* (22.10)	0.209ns (1.70)
	L*C*D	0.453ns (3.60)	0.394ns (3.20)	0.397ns (3.20)	1.463ns (10.90)	1.410ns (10.50)	0.601ns (4.80)

The L represents the micropore group spacing, the C represents the capillary arrangement density, the D represents the different planting seasons of tomato, the data are all average ± standard deviation in the table, the bracketed number is the factor relative contribution%, the same below. The ACE represents ACE index of soil bacterial, CHAO represents CHAO index of soil bacterial, COVERAGE represents COVERAGE index of soil bacterial, SHANNON represents SHANNON index of soil bacterial, SIMPSON represents SIMPSON index of soil bacterial, SOBS represents SOBS index of soil bacterial. Different letters in the same line meant significant difference at 0.05 level, *: P<0.05, **: P<0.01, ns: P>0.05.

Compared with L2, the ACE, CHAO, SHANNON, and SOBS of spring tomato and autumn tomato treated with L1 were higher. With the decrease of C, the ACE, CHAO, SHANNON, SIMPSON and SOBS of soil bacterial with spring tomato and autumn tomato showed a decreasing trend. The diversity of soil bacterial community structure in spring tomato was lower than that in autumn tomato.

#### 3.1.2 Soil bacterial community structure species composition

**Figure 6:**
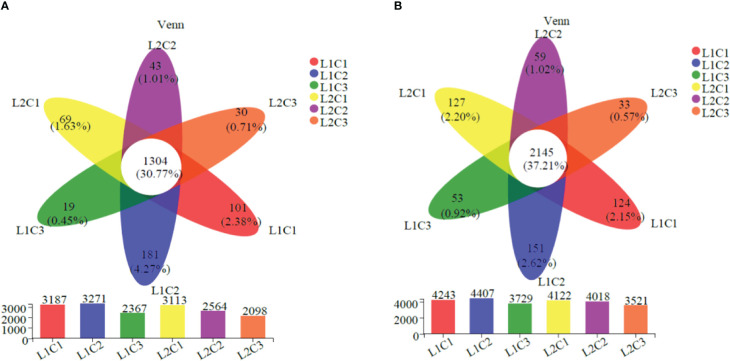
OTUs classification of soil bacteria. **(A)** Spring **(B)** Autumn.

As can be seen from **Figure 6**, there are 1304 and 2145 identical OTUs in the soil bacteria of spring tomato and autumn tomato in the six treatments, accounting for 30.77% and 37.21% of the total OTUs. Single factor significant analysis showed that the total number of soil bacteria in spring tomato and autumn tomato treated with L1C2 was the highest at the OTUs classification level (3271 and 4407). Compared with L2, the total number of soil bacteria in spring tomato and autumn tomato treated with L1 increased by 13.50% and 6.16% at the OTUs classification level. With the decrease of C, the total number of soil bacteria in spring tomato and autumn tomato decreased at the OTUs classification level. Compared with C3, the total number of soil bacteria in C2 spring tomato and autumn tomato increased by about 30.68% and 16.21% at the OTUs classification level.

**Figure 7:**
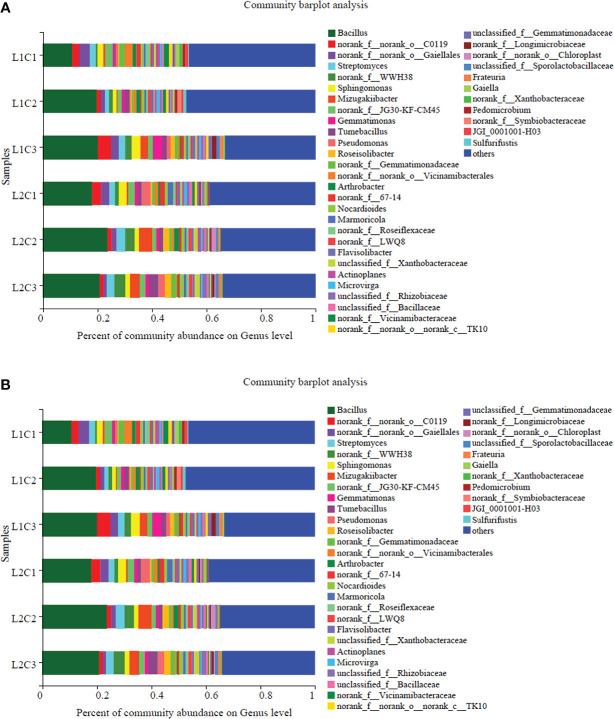
Taxonomic level of soil bacteria. **(A)** Spring **(B)** Autumn.

From **Figure 7**, it can be seen that the dominant bacterial populations in spring tomato soil at the genus level of soil bacteria mainly include *Bacillus* (10.71%-23.68%), *Streptomyces* (1.44%-3.17%), *Sphingomonas* (1.40%-3.21%); the soil of autumn tomato mainly includes *Sphingomonas* (6.07%-10.68%), *Bacillus* (2.55%-6.85%) and *Gemmatimonas* (1.75%-2.65%). *Bacillus* is a common genus of spring tomato and autumn tomato soil. With the decrease of C, the abundance of *Bacillus* in spring and autumn tomato increased first and then decreased. Compared with L2, the abundance of *Bacillus* with spring and autumnin L1 treatment increased by 23.50% and 76.17% at genus level.

### 3.2 Soil bacterial nitrogen metabolism functional gene abundance and soil bacterial phosphorus metabolism functional gene abundance

From **Table 4**, it can be seen that the L has a significant effect on the soil bacterial nitrogen metabolism functional gene abundance (GAN) and soil bacterial phosphorus metabolism functional gene abundance (GAP). The contribution of L to the GAN and GAP in tomato were 18.90%, 21.80%, respectively. The C had a significant effect on the soil bacterial nitrogen fixation functional gene abundance (NFFA), soil bacterial nitrification functional gene abundance (NFA), soil bacterial nitrification functional gene abundance (DFA), GAN and GAP. The relative contribution of capillary arrangement density to the NFFA, NFA, GAN and GAP in tomato soil were 72.60%, 63.20%, 30.90%, 60.90% and 48.20%, respectively. The D had a significant effect on the NFFA, NFA, DFA, GAN and GAP. The relative contribution of D to the NFFA, NFA, DFA, GAN and GAP in tomato soil were 96.60%, 96.70%, 86.70%, 95.30% and 93.30%, respectively. The GAN and GAP in L1C2 treatment were not significantly lower than that in L1C1 and L2C1, but significantly higher than that in L1C3, L2C2 and L2C3 treatments, indicating that L1C2 treatment had higher nitrogen and phosphorus metabolism functional gene abundance, which could promote soil nitrogen and phosphorus cycle transformation.

**Table 4:** Comparative analysis functional genes related to N and P metabolism of soil bacteria.

	Treatment	NFFA	NFA	DFA	GAN	GAP
Spring	L1C1	7968 ± 1184.68a	4146.33 ± 397.81a	10190 ± 734.72bc	22304.33 ± 2125.17a	164247 ± 4734.48a
	L1C2	6377.33 ± 1372.51ab	4053 ± 218.09ab	10862.67 ± 280.9ab	21293 ± 2111.14a	151945.67 ± 6087.11a
	L1C3	3936 ± 145.14c	3527.33 ± 276.1bc	9354 ± 193.45c	16817.33 ± 443.6b	113045.67 ± 5058.62c
	L2C1	5288.67 ± 1201.81bc	4360.67 ± 276.8a	11608.67 ± 658.01a	21258 ± 4228.28a	152421.67 ± 15321.09a
	L2C2	3671.67 ± 101.19c	3374.33 ± 377.31c	9761.67 ± 307.11bc	16807.67 ± 1167.53b	137102 ± 6061.43b
	L2C3	3722.67 ± 248.09c	3343 ± 340.74c	9295.67 ± 1164.67c	16361.33 ± 1054.67b	112817.67 ± 5730.07c
Autumn	L1C1	21089 ± 969.35a	10654.67 ± 589.55a	22881.33 ± 835.97a	54625 ± 1747.79a	397943.33 ± 19602.93a
	L1C2	19958.33 ± 1954.3ab	9289 ± 965.06ab	20197 ± 2016.25a	49444.33 ± 3855.53ab	371200.33 ± 16870.22a
	L1C3	16616 ± 1842.29b	8918 ± 508.66b	18578.33 ± 4332.23a	44112.33 ± 5241.44b	333575 ± 70090.14ab
	L2C1	20697.33 ± 1091.38a	8839.67 ± 1253.06b	21807.33 ± 3075.77a	51344.33 ± 3767.77ab	357470.33 ± 9445.11a
	L2C2	19851 ± 3432.89ab	9644.67 ± 300.9ab	20517.67 ± 4273.95a	50013.33 ± 7947.17ab	337817 ± 73672ab
	L2C3	11662 ± 1285.12a	6339 ± 444.66c	16598 ± 3757.27a	34599 ± 5110.82c	260776.33 ± 45050.68b
F-value	L	13.273** (35.60)	16.422** (40.60)	0.270ns (1.10)	5.578* (18.90)	6.685* (21.80)
	C	31.828** (72.60)	20.601** (63.20)	5.375* (30.90)	18.660** (60.90)	11.158** (48.20)
	D	676.695** (96.60)	712.845** (96.70)	156.417** (86.70)	481.999** (95.30)	334.246** (93.30)
	L*C	0.544ns (4.30)	3.341* (21.80)	0.188ns (1.50)	0.577ns (4.60)	0.118ns (1.00)
	L*D	0.002ns (0.01)	8.589** (26.40)	0.396ns (1.60)	0.654ns (2.70)	3.183ns (11.70)
	C*D	8.023** (40.10)	6.266** (34.30)	1.348ns (10.10)	4.509* (27.30)	0.881ns (6.80)
	L*C*D	5.616** (31.9)	7.953** (39.90)	0.593ns (4.70)	2.513ns (17.30)	0.549ns (4.40)

The L represents the micropore group spacing, the C represents the capillary arrangement density, the D represents the different planting seasons of tomato, the data are all average ± standard deviation in the table, the bracketed number is the factor relative contribution%, the same below. The NFFA represents the soil bacterial nitrogen fixation functional gene abundance, the NFA represents the soil bacterial nitrification functional gene abundance, the DFA represents the soil bacterial denitrification functional gene abundance, the GAN represents the soil bacterial nitrogen metabolism functional gene abundance, GAP represents the soil bacterial phosphorus metabolism functional gene abundance. Different letters in the same line meant significant difference at 0.05 level, *: P<0.05, **: P<0.01, ns: P>0.05.

Compared with L2, the NFFA, NFA, soil bacterial denitrification functional gene abundance (DFA), GAN and GAP in spring tomato and autumn tomato soil treated with L1 increased by 44.14% and 10.44%, 5.85% and 16.27%, -0.85% and 4.64%, 11.00% and 8.99%, 6.69% and 15.34%, respectively. With the decrease of C, the NFFA, NFA, DFA, GAN and GAP in spring tomato and autumn tomato soil increased first and then decreased. The NFFA, NFA, DFA, GAN and GAP in C2 of spring tomato and autumn tomato soil was significantly higher than that in C3 about 31.21% and 40.78%, 8.11% and 24.10%, 10.59% and 15.74%, 14.83% and 26.36%, 27.97% and 19.29%, respectively. The abundance of soil bacterial nitrogen metabolism and phosphorus metabolism functional genes in spring tomato was lower than that in autumn tomato.

### 3.3 Effects of different treatments on tomato roots in greenhouse

It can be seen from **Table 5** that the L had significant effects on total root length (RL), total number of root tips (RT) and root activity (RA) of tomato, in which the relative contributions of L to RL, RT and RA of tomato are 11.80%, 12.60% and 11.90%, respectively. The C had a significant effect on RL, total root surface area (RS), total root volume (RV), RT, total root bifurcation number (RB) and RA of tomato (*P ≤* 0.05). The relative contribution of C to RL, RS, RV, RT, RB and RA of tomato was 16.10%, 16.40%, 16.10%, 18.60%, 17.50% and 13.20%, respectively. The D had a significant effect on RL, RS, RV, RT, RB and RA of spring tomato and autumn tomato (*P ≤* 0.05). The relative contribution of D to RL, RS, RV, RT, RB and RA of tomato was 23.00%, 22.60%, 13.20%, 10.30%, 9.70% and 13.20%, respectively.

**Table 5:** Root morphology of tomato in greenhouse.

	Treatment	RL cm/plant	RS cm^2^/plant	RV cm^3^/plant	RT	RB	RA mg/g/h
Spring	L1C1	363.63 ± 28.95ab	119.5 ± 25.65a	3.3 ± 1.36a	831.11 ± 131.75a	1774.44 ± 591.7ab	2.31 ± 0.25ab
	L1C2	369.43 ± 16.2a	121.18 ± 24.56a	3.42 ± 1.38a	838 ± 56.99a	1984.44 ± 485.65a	2.53 ± 0.61a
	L1C3	325.4 ± 47.93ab	98.44 ± 17.29ab	2.42 ± 0.55a	706.89 ± 158.88b	1312.33 ± 479.37bc	2.05 ± 0.37b
	L2C1	359.26 ± 53.15ab	117.46 ± 26.01ab	3.27 ± 1.33a	816.89 ± 220.85a	1761.56 ± 641.17ab	2.29 ± 0.54ab
	L2C2	353.89 ± 49.96ab	116.31 ± 20.29ab	3.27 ± 1a	807.67 ± 111.26ab	1649.67 ± 495.41abc	2.2 ± 0.49ab
	L2C3	318.3 ± 53.25b	95.89 ± 18.27b	2.3 ± 0.61b	663.89 ± 158.1b	1162.89 ± 350.5c	1.87 ± 0.35b
Autumn	L1C1	317.31 ± 35.94a	96.4 ± 12.28ab	2.48 ± 0.94ab	824.56 ± 102.27a	1503.33 ± 443.89a	1.41 ± 0.25a
	L1C2	322.82 ± 39.36a	102.63 ± 16.34a	2.82 ± 0.89a	809.89 ± 125.52a	1454 ± 379.42ab	1.45 ± 0.56a
	L1C3	294.9 ± 39.84ab	83.53 ± 19.84b	1.87 ± 0.55b	684.67 ± 106.29ab	1216.22 ± 260.74ab	1.16 ± 0.22a
	L2C1	310.01 ± 23.53ab	89.07 ± 12.09ab	2.24 ± 0.81ab	822.33 ± 168.11a	1334.44 ± 326.83ab	1.35 ± 0.21a
	L2C2	315.53 ± 45.3a	94.49 ± 23.15ab	2.55 ± 0.89ab	764.78 ± 176.69ab	1388.89 ± 293.34ab	1.31 ± 0.22a
	L2C3	271.91 ± 47.29b	80.39 ± 18.3b	1.82 ± 0.49b	664.89 ± 103.06b	1132.22 ± 273.35b	1.14 ± 0.26a
F-value	L	3.803* (11.80)	1.476ns (1.50)	0.62ns (0.60)	3.899** (12.60)	2.632ns (2.70)	3.837* (11.90)
	C	9.183** (16.10)	9.447** (16.40)	9.232** (16.10)	10.999** (18.60)	10.196** (17.50)	7.280** (13.20)
	D	28.645** (23.00)	27.968** (22.60)	14.637** (13.20)	4.327* (10.30)	10.349** (9.70)	147.428** (60.60)
	L*C	0.122ns (0.20)	0.075ns (0.20)	0.041ns (0.10)	0.109ns (0.20)	0.154ns (0.30)	0.589ns (1.00)
	L*D	0.048ns (0.10)	0.157ns (0.20)	0.055ns (0.10)	0.016ns (0.01)	0.127ns (0.10)	0.459ns (0.50)
	C*D	0.114ns (0.20)	0.624ns (1.30)	0.446ns0.90)	0.146ns0.30)	1.539ns0.31)	0.435ns (0.90)
	L*C*D	0.189ns (0.40)	0.031ns (0.10)	0.046ns (0.10)	0.043ns (0.10)	0.539ns (0.11)	0.274ns (0.60)

The L represents the micropore group spacing, the C represents the capillary arrangement density, the D represents the different planting seasons of tomato, the data are all average ± standard deviation in the table, the bracketed number is the factor relative contribution%, the same below. The RL represents the total root length, the RS represents the total root surface area, the RV represents the total root volume, the RT represents the total number of root tips, the RB represents the total root bifurcation number, the RA represents the root activity. Different letters in the same line meant significant difference at 0.05 level, *: P<0.05, **: P<0.01, ns: P>0.05.

Compared with L2, the RL, RS, RV, RT, RB and RA of spring tomato and autumn tomato in L1 increased by 2.62% and 4.19%, 2.87% and 7.05%, 3.45% and 8.49%, 3.83% and 2.98%, 10.87% and 8.25%, 8.31% and 5.94%, respectively. With the decrease of C, the RL, RS, RV, RT, RB and RA of spring tomato and autumn tomato increased first and then decreased. The RL, RS, RV, RT, RB and RA of spring tomato and autumn tomato in C2 treatment were about 1.06% and 1.76%, 1.22% and 6.28%, 1.76% and 14.07%, 0.14% and 4.39%, 2.77% and 0.18%, 2.67% and 1.04% higher than those in C1 treatment. It was also higher than C3 treatment by about 12.37% and 12.62%, 22.21% and 20.25%, 41.73% and 45.63%, 20.05% and 16.68%, 46.82% and 21.05%, 20.42% and 20.23%, respectively. The root morphological development of spring tomato was better than that of autumn tomato.

### 3.4 Effects of different treatments on yield and water use efficiency of greenhouse tomato

**Figure 8:**
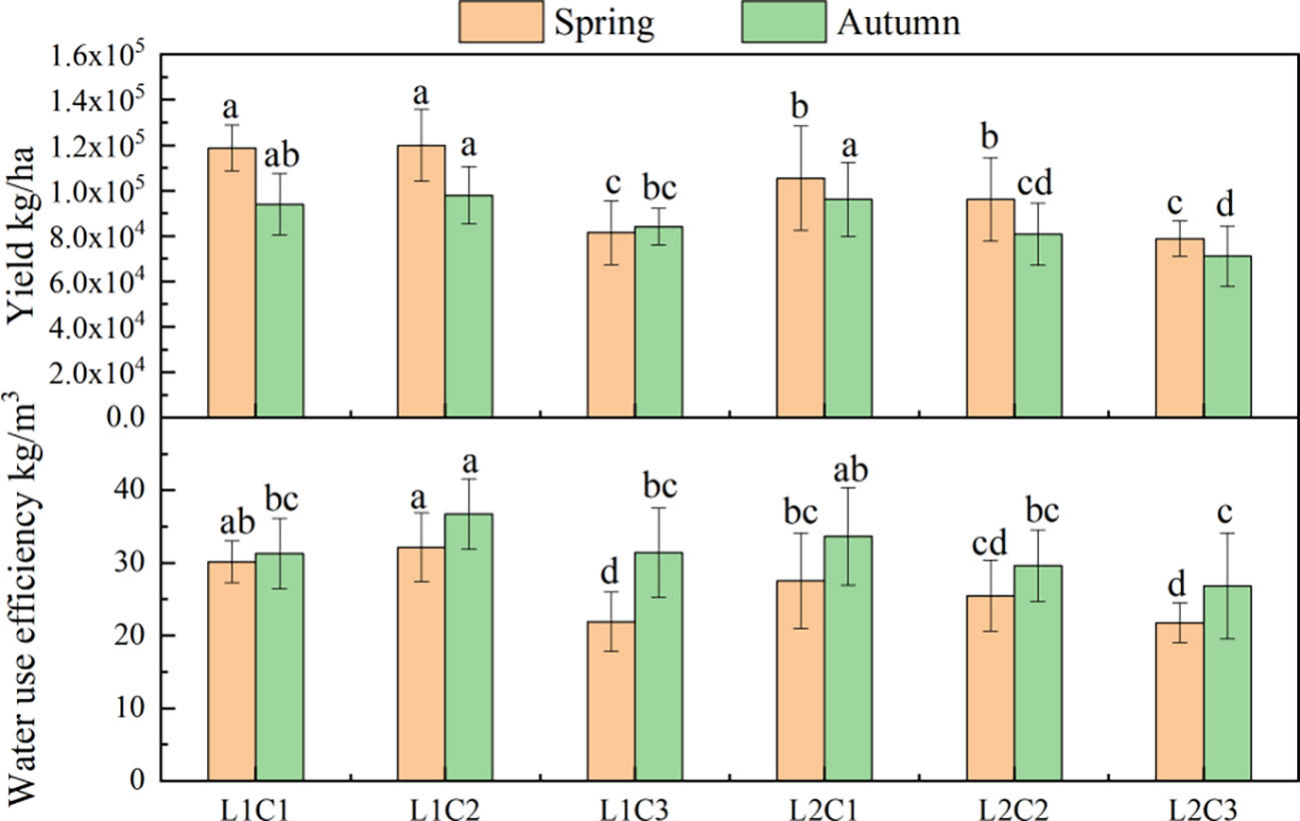
Yield and water use efficiency of tomato. The data are all average±standard deviation in the figure, different letters in the same column meanS significant difference at 0.05 level, the same as blow.

It can be seen from **Figure 8** that the L and C have significant effects on yield (Y) and water use efficiency (WUE) of spring and autumn tomatoes. Compared with L2, the Y and WUE of spring tomato and autumn tomato in L1 treatment increased by 14.15% and 11.27%, 12.64% and 10.35%, respectively. With the decrease of C, the Y and WUE of spring tomato and autumn tomato showed an increasing trend. The Y and WUE of C2 spring tomato and autumn tomato were significantly higher than those of C3 by about 34.76% and 15.23%, 31.94% and 13.91%, respectively.

### 3.5 Correlation of tomato soil bacterial community, root system and yield

**Figure 9:**
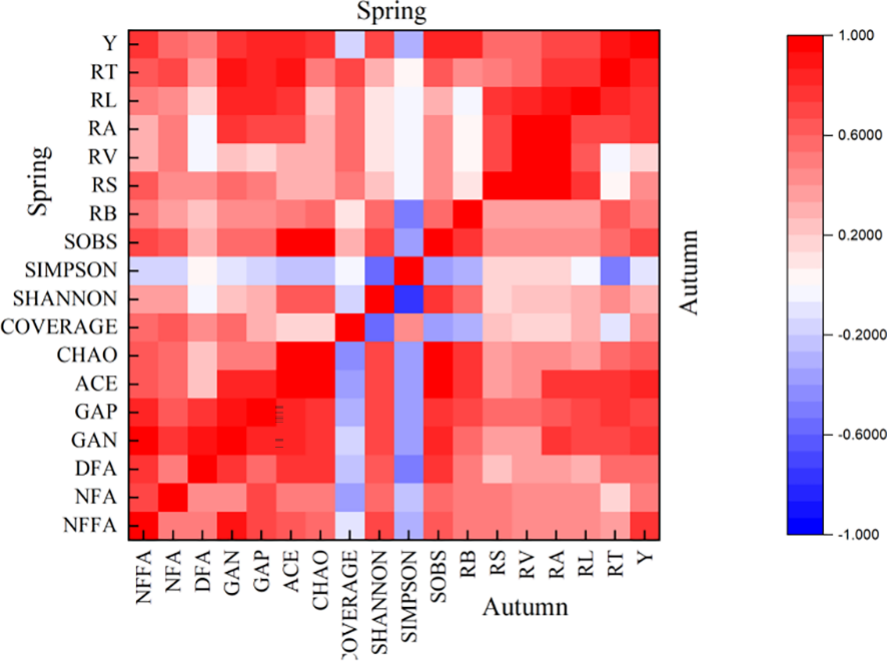
Correlation between tomato soil bacterial community, root system and yield. Spring represents the correlation between the indicators of spring tomato; Autumn represents the correlation between the indicators of autumn tomato. The NFFA represents the soil bacterial nitrogen fixation functional gene abundance, the NFA represents the soil bacterial nitrification functional gene abundance, the DFA represents the soil bacterial denitrification functional gene abundance, the GAN represents the soil bacterial nitrogen metabolism functional gene abundance, GAP represents the soil bacterial phosphorus metabolism functional gene abundance. The ACE represents ACE index of soil bacterial, CHAO represents CHAO index of soil bacterial, COVERAGE represents COVERAGE index of soil bacterial, SHANNON represents SHANNON index of soil bacterial, SIMPSON represents SIMPSON index of soil bacterial, SOBS represents SOBS index of soil bacterial. The RL represents the total root length, the RS represents the total root surface area, the RV represents the total root volume, the RT represents the total number of root tips, the RB represents the total root bifurcation number, the RA represents the root activity. The Y represents the yield.

Based on Pearson’s two-tailed test, the indexes with better correlation among soil bacterial community, root system, and yield were screened out. Correlation analysis (**Figure 9**) showed that ACE, GAN, GAP RL, RT and RA were positively correlated with tomato yield, indicating that there was a positive interaction between soil bacterial community and root system. A stable bacterial community and good root morphological development positively promoted the increase of tomato yield. It was also found that the ACE, GAN and GAP had the highest positive correlation with the RT in the root index (0.758 and 0.870, 0.704 and 0.875, 0.756 and 0.853). In order to further quantitatively describe how the soil bacterial community affects tomato roots and thus affects tomato yield, we selected RT, which has the highest correlation with ACE, GAN and GAP, as a bridge. The relationship between ACE, GAN, GAP and RT, and the relationship between RT and yield were quantitatively described by regression analysis, respectively. The analysis results are shown in **Figure 9**.

**Figure 10:**
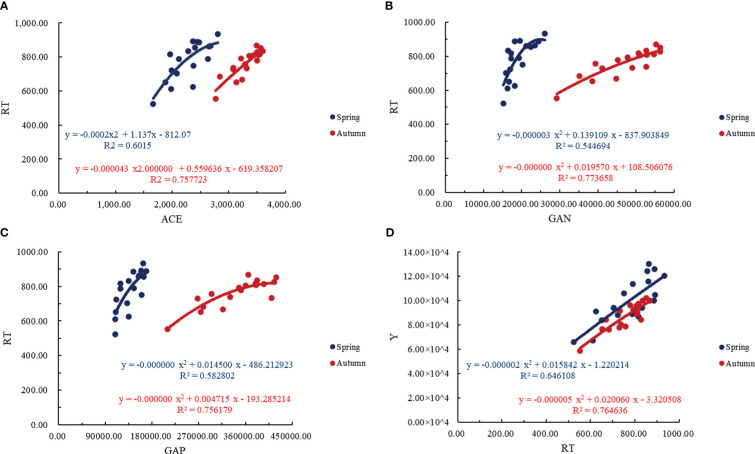
Quantitative analysis of tomato soil bacterial community, root system and yield in greenhouse. **(A)** represents the regression analysis of ACE index of soil bacterial and total number of root tips, **(B)** represents the regression analysis of soil bacterial nitrogen metabolism functional gene abundance and total number of root tips, **(C)** represents the regression analysis of soil bacterial phosphorus metabolism functional gene abundance and total number of root tips, **(D)** represents the regression analysis of yield and total number of root tips. The ACE represents ACE index of soil bacterial, the GAN represents the soil bacterial nitrogen metabolism functional gene abundance, GAP represents the soil bacterial phosphorus metabolism functional gene abundance, the RT represents the total number of root tips, the Y represents the yield.

From **Figure 10**, it can be seen that the ACE and RT showed a quadratic parabolic curve relationship, in which the determination coefficient R^2^>0.6015, indicating that the ACE in the regression model can explain 60.15% of the change of tomato RT. The GAN and GAP also showed a quadratic parabolic curve relationship with the RT of tomato. The determination coefficient R^2^ of the regression equation between the GAN and the RT of tomato was higher than that of the determination coefficient R^2^ of the regression equation between the GAP and the RT of tomato, indicating that the GAN had a greater impact on the RT of tomato. The relationship between the RT of tomato and the yield was a quadratic parabolic curve, and the coefficient of determination R^2^>0.6461, indicating that the RT of tomato in the regression model could explain 64.61% of the change in yield of tomato. The RT of tomato could be used to estimate the yield, and the tomato production potential in this area could be indirectly evaluated by soil bacterial community.

## 4 Discussion

### 4.1 The layout measures regulate the soil bacterial community of greenhouse tomato

Previous studies have shown that under drought stress, plants will reduce the supply of carbon sources for soil bacteria, and sustainably reduce the diversity and richness of bacterial community structure (de Vries et al., 2018; Zeng et al., 2019). It was found that the diversity of bacterial community structure in tomato soil treated with L1 was higher than that of L2. It may be due to the high soil volumetric water content and strong soil aeration in the tillage layer with a micropore group spacing of 30 cm. The average soil volumetric water content in the 0-40 cm soil layer of spring tomato and autumn tomato irrigated by L1 treatment was about 2.18% and 3.61% higher than that of L2, and the soil water filling porosity was only 0.92 and 0.93 times that of L2. The higher water vapor environment of the soil can reduce the limitation of drought stress and hypoxia stress on soil bacterial community (Hartmann and Six, 2022; Tang et al., 2022). This conclusion is consistent with the study of Qu (Qu et al., 2022) and Alekhina (Alekhina et al., 2001) that drought stress reduces the diversity of soil bacterial community structure. The L1 had higher soil GAN of tomato than L2. It may be due to the fact that the L1 has higher soil volume moisture content and strong soil ventilation, increase root exudates and root nitrogenase activity and increase the process of above-ground carbon assimilation of tomato. Further increase the transport of carbohydrates to the underground part of tomato (root), higher root morphological development can provide more living substrates for soil bacteria (Pathania et al., 2020; Tiziani et al., 2022; Wang et al., 2023). At the same time, the rhizobia in tomato soil were greatly affected by soil moisture, which easily affected the identification and infection of host plants, and finally promoted the abundance of functional genes of soil bacterial nitrogen metabolism under L1 treatment irrigation (Mantovani et al., 2014).

The C can change the composition of microbial community by changing the wet/dry conditions of soil, thus changing the mineralization rate of soil nutrients and affecting the absorption capacity of crop roots to nutrients (Wang Z. et al., 2022; Wang et al., 2021). This study found that the diversity of bacterial community structure in tomato rhizosphere soil of C2 treatment was significantly higher than that of C3 treatment. It may be because the soil water availability per unit irrigated plough layer of C2 treatment is significantly higher than that of C3 by 6.67% and 6.69%, respectively. Stress effects of lower soil moisture increase on soil microorganisms (Alekhina et al., 2001). At the same time, the irrigation control area of C3 is larger, the irrigation amount in the plot is larger, and the local soil moisture experience is longer and higher, which is easy to increase the effect of low oxygen stress on soil microorganisms. The uneven distribution of soil moisture per unit area of C3 promotes the soil organisms in the rhizosphere of tomato under low water and low oxygen stress. Because of the existence of the filtration mechanism of the living environment, some bacteria in the soil are eliminated (Xu et al., 2020; Yang et al., 2020). The conclusion of this study is consistent with the conclusion of Wang (Wang, 2017) that the capillary density of one tube and two rows of drip irrigation promotes the diversity of microbial community structure in greenhouse tomato rhizosphere soil, and it is also consistent with the conclusion that the excessive number of dry-wet cycles of Jiao (Jiao et al., 2022) reduces the diversity of soil bacterial structure. The study also found that the abundance of nitrogen and phosphorus metabolism functional genes in C2 treated tomato rhizosphere soil was significantly higher than that in C3. It may be due to the decrease of soil litter concentration and carbon decomposition under C3, the substrate concentration of soil nitrogen and phosphorus bacteria decreased, and then decreased the abundance of nitrogen and phosphorus metabolism genes of soil bacteria (Jin et al., 2013; Zhang J. et al., 2022; Ullah et al., 2023).

### 4.2 The layout measures regulate the root system of greenhouse tomato

The effect of C change of MSPF on soil wetted body is similar to that of dripper spacing change (Chen et al., 2010; Zhang M. et al., 2022). There is a phenomenon that wetting fronts intersect between two adjacent groups of micropores on the pipe. The larger the distance is, the smaller the ratio of horizontal migration distance to vertical migration distance of soil moisture wetting front is, which is not conducive to the overall deep migration of soil moisture between two groups of micropores on the capillary. The volumetric water content and irrigation uniformity of plough layer per unit area will decrease (Ould Mohamed El-Hafedh et al., 2001; Sun and Wang, 2007; Del Vigo et al., 2020). The average soil volumetric water content in 0-40 cm soil layer of spring tomato and autumn tomato in L1 was 2.18% and 3.61% higher than that in L2. The higher soil volumetric water content in the plough layer was beneficial to the development of crop root morphology (Abdelhafeez et al., 1975; Silveira et al., 2020). This may be one of the reasons why the root morphological development and root activity of spring tomato and autumn tomato under L1 were higher than those under L2. The conclusion of this study is consistent with Zhang (Zhang S. et al., 2022) who found that the uniformity of soil nutrient distribution of drip irrigation 30 cm dripper spacing is higher than that of 50 cm dripper spacing, which is conducive to the development of apple root morphology.

This study found that too high or too low capillary density was not conducive to tomato root morphological development. It may be due to the increase of the outflow cross section per unit area in the test area when the C is too high (C1), which is easy to be saturated in shallow soil, form water accumulation layer on the surface, restrict the exchange of soil air and external gas, produce hypoxia stress and restrict root growth (Aixia et al., 2022; Junhao et al., 2022). When the C was too low (C3), the outflow cross section per unit area in the experimental plot decreased, the soil moisture distribution per unit area was uneven, the deep leakage increased, which led to the decrease of soil moisture in tomato tillage layer. The average soil volumetric water content in the cultivated layer (0-40 cm) of spring tomato and autumn tomato treated with C2 was significantly higher than that of C3 by about 6.67% and 6.69%, respectively. Drought stress limits root morphological development (Enciso et al., 2007; Samoy-Pascual et al., 2022; Fan et al., 2023). The conclusion of this study is consistent with the conclusion of Liu’s (Liu et al., 2019) study that too high or too low drip pipe spacing is not conducive to the development of alfalfa root morphology under drip irrigation.

### 4.3 Layout measures to regulate bacterial community and root interaction in tomato soil

Previous studies have found that the difference in soil water distribution caused by irrigation can significantly affect soil bacterial community, crop root morphological development, and increase or decrease soil carbon, nitrogen and phosphorus metabolic activity (Wang et al., 2021; Wang J. et al., 2022). It was found that there was a significant positive correlation between the diversity of bacterial community structure soil, the abundance of soil nitrogen and phosphorus metabolic genes in tomato rhizosphere and the RA, RL and RT of tomato roots. It may be that the RA, RL and RT determine the distribution and structure of tomato root system in soil. The high values of these indexes can improve the ability of plant roots to obtain soil moisture and nutrients, to colonize roots and enhance and enhance the strength of root-microorganism interaction. On the contrary, the diversity and abundance of soil bacterial community structure are high, which is conducive to the mineralization of soil organic matter, more soil nutrients are absorbed and utilized by plants, and the development of tomato root morphology is promoted (Leng et al., 2022; Andrade et al., 2023). It is consistent with the conclusion that Wang (Wang et al., 2017) drip irrigation crop root length is positively correlated with soil bacterial community structure diversity and yield, which is consistent with the conclusion that Nazir (Nazir et al., 2021) drip irrigation rape root activity, length and yield are positively correlated.

Previous studies have found that there is an interactive relationship between tomato root morphological development and soil microorganisms (Wang J. et al., 2022). In this study, the root morphological development of L1C2 treatment is better, which may be one of the reasons why the soil bacterial community of L1C2 treatment is superior to the other five treatments. In addition, we found that the RT was the key factor of tomato soil bacteria and tomato root interaction and played an important role in enhancing the positive interaction between soil bacteria and roots (**Figure 10**). The L1C2 significantly increased the RT of tomato, thus promoting the benign interaction between bacteria and roots in tomato soil. The effect of the layout measures of MSPF on the interaction between soil bacteria community and roots in tomato will inevitably affect the tomato yield. In this study, we found that the RT and ACE had the greatest influence on tomato yield under the regulation of MSPF. It may be that the layout measures of MSPF directly affects the RT and soil bacteria, and indirectly affects the interaction between tomato root system and soil bacteria through soil moisture, and regulates tomato yield directly or indirectly. In this study, the soil moisture distribution in the root zone of tomato treated with L1C2 was uniform, and the RT and ACE were significantly increased, which promoted the tomato yield under L1C2 better than other treatments.

## 5 Conclusion

By exploring the layout measures of MSPF to regulate the soil bacterial community and root morphological development of tomato in greenhouse, it was found that the diversity and abundance of soil bacterial community structure of spring tomato and autumn tomato in L1 were higher than those of L2. Among them, the total number of OTUs classification of soil bacteria in spring tomato and autumn tomato with L1 was higher than that of L2 by about 13.50% and 6.16%, respectively, and the ACE in spring tomato and autumn tomato with L1 was higher than that of L2 about 6.90% and 8.19%, respectively. The GAN and GAP in spring tomato and autumn tomato soil with L1 was higher than that of L2 about 11.00% and 8.99%, 6.69% and 15.34%, respectively. As a result, the yield and water use efficiency of spring tomato and autumn tomato treated with L1 were higher than those of L2 by about 14.15% and 11.27%, 12.64% and 10.35%, respectively. With the decrease of C, the diversity of soil bacterial community structure decreased significantly. Among them, the ACE and total number of OTUs classification of soil bacteria with C2 was significantly higher than that of C3 about 27.59% and 9.85%, 30.68% and 16.21%, respectively. The GAN and GAP decreased with the decrease of C. For example, the GAN and GAP in soil of spring tomato and autumn tomato with C2 was significantly higher than that of C3 about 14.83% and 26.36%, 27.97% and 19.29%, respectively. Lower soil bacterial community structure diversity and soil bacterial nitrogen and phosphorus metabolism functional gene abundance reduced greenhouse tomato soil nitrogen and phosphorus cycle. As a result, the yield and water use efficiency of spring and autumn tomatoes with C2 were significantly higher than those of C3 by 34.76% and 15.23%, 31.94% and 13.91%, respectively. Pearson two-tailed test and regression analysis showed that there was a positive interaction between soil bacterial community and root morphological development. The relationship between soil bacterial community and RT was a quadratic curve, and the relationship between RT and yield was also quadratic curve, indicating that the RT of tomato could be used to estimate the yield, and the tomato production potential in this area could be indirectly evaluated by soil bacterial community. This study provides a reference for regulating tomato root system and soil microbial interaction and increasing tomato yield by optimizing the layout measures of MSPF.

## Data availability statement

The data presented in the study are deposited in the figshare repository, https://figshare.com/articles/dataset/The_Layout_Measures_of_Micro-sprinkler_Irrigation_under_Plastic_Film_Regulate_Tomato_Soil_Bacterial_Community_and_Root_System/21818610.

## Author contributions

MZ wrote and revised the manuscript. YL participated in the experiments. NX, HY, FG, and JL collected the materials and analyzed the data. YL, ZZ and MZ conceived and designed the research. All authors contributed to the article and approved the submitted version.
